# Bayesian Estimation of Vancomycin Exposure and Population Pharmacokinetics in Obese Patients

**DOI:** 10.3390/antibiotics15050485

**Published:** 2026-05-11

**Authors:** Ha-Jin Chun, Hyeon Gyeom Choi, Eun Sook Bang, Jin Ok Kyun, Young Rong Kim, Eun Jin Kim, So Hee Kim

**Affiliations:** 1 Department of Pharmacy, Ajou University Hospital, Suwon 16499, Republic of Korea; 2 College of Pharmacy, Ajou University, Suwon 16499, Republic of Korea; 3 Research Institute of Pharmaceutical Science and Technology, Ajou University, Suwon 16499, Republic of Korea; 4 Department of Diagnostic & Biomedical Sciences, School of Dentistry, The University of Texas Health Science Center at Houston, Houston, TX 77054, USA; 5 Department of Infectious Diseases, Ajou University School of Medicine, Suwon 16499, Republic of Korea; 6 Department of Biohealth Regulatory Science, Graduate School of Ajou University, Suwon 16499, Republic of Korea

**Keywords:** vancomycin, therapeutic drug monitoring, obese patients, Bayesian program, population pharmacokinetics

## Abstract

**Background/Objectives**: This study compared the pharmacokinetic (PK)/pharmacodynamic (PD) targets of vancomycin in obese patients using two Bayesian programs to evaluate the accuracy and bias of these estimates. Additionally, population pharmacokinetics in obese patients were compared with those in the general patient population. **Methods**: Medical records of obese adults [body mass index (BMI) ≥ 30 kg/m^2^] treated with vancomycin at Ajou University Hospital between 2010 and 2017 were retrospectively reviewed. Patients with peak (*C*_peak_) and trough (*C*_trough_) concentrations were included. Vancomycin area under the plasma concentration-time curve (AUC) and *C*_trough_ were estimated using Capcil (ver 6.31) and MwPharm (ver 2.3.1.89) software. Accuracy and bias were analyzed, and population PK parameters were evaluated. **Results**: A total of 149 cases were analyzed. AUC estimates from one-point sampling using Capcil were significantly lower than the reference values, whereas MwPharm produced estimates closer to the reference with higher accuracy and lower bias. *C*_trough_ estimates from one-point sampling showed high accuracy for both programs. Obese patients exhibit lower volume of distribution, higher total body clearance, and shorter elimination half-life than the general patient population. **Conclusions**: Vancomycin pharmacokinetics differ in obese patients. Bayesian analysis using MwPharm showed AUC and *C*_trough_ estimates that were closer to the reference values and exhibited lower bias than those obtained with Capcil in this dataset. These findings may support more informed dosing decisions for vancomycin in obese patients.

## 1. Introduction

Over the past decade, the prevalence of obesity has increased worldwide, presenting an increasing clinical challenge. In Korea, the prevalence of obesity among adult women has remained relatively stable at approximately 27%, whereas that among men has risen substantially from 35.1% in 2011 to 46.3% in 2021 [1]. Obesity is of particular concern in clinical pharmacokinetics because it can alter drug distribution and elimination, thereby complicating dosing strategies and increasing the risk of under- or overexposure [2,3].

For more than five decades, vancomycin—a glycopeptide antibiotic—has been the cornerstone therapy for severe infections caused by methicillin-resistant *Staphylococcus aureus* (MRSA) [4,5,6]. Early pharmacodynamic (PD) evaluations of vancomycin focused on the percentage of time for which drug concentrations exceeded the minimum inhibitory concentration (MIC). However, subsequent experimental and clinical studies demonstrated that the ratio of the area under the plasma concentration–time curve (AUC) from 0 to 24 h (AUC_0–24h_) to MIC (AUC_0–24h_/MIC) is a more robust predictor of vancomycin efficacy in MRSA infections [7,8]. Accordingly, the 2009 consensus guidelines recommended AUC/MIC as the preferred pharmacokinetic (PK)/PD index, with trough plasma concentrations (*C*_trough_) of 15–20 μg/mL proposed as a surrogate marker for achieving adequate exposure [4,9].

However, subsequent evidence has shown that *C*_trough_ is an imperfect surrogate for AUC and may be associated with an increased risk of nephrotoxicity without a corresponding improvement in clinical efficacy [10,11]. Consequently, the 2020 revised guidelines now recommend direct AUC-guided dosing, targeting an AUC of 400–600 mg·h/L, while assuming an MIC of 1 mg/L as determined by broth microdilution [11,12,13]. This shift has been widely recognized as a major paradigm change in therapeutic drug monitoring (TDM) of vancomycin.

Accurate estimation of vancomycin AUC can be achieved using first-order PK equations with two steady-state plasma concentrations or Bayesian estimation approaches that integrate patient-specific covariates and sparse sampling data [12,14,15]. Bayesian software programs offer practical advantages in clinical settings by allowing flexible sampling strategies. However, their performance inherently depends on the underlying population PK models and prior distributions embedded within each program [11,14,16,17].

Vancomycin is a hydrophilic drug whose distribution is more closely related to total body water than to adipose tissue [11]. In obese patients, a rise in body weight does not result in proportional expansion of the volume of distribution (*V*_d_), which potentially leads to systematic bias when standard population models are applied [18,19,20]. Although several Bayesian programs are routinely used, their reliability in estimating the AUC and *C*_trough_ in obese patients remains insufficiently validated.

The present study aimed to evaluate the accuracy and bias of vancomycin AUC and *C*_trough_ estimates derived from one- and two-point blood sampling using two Bayesian software programs in obese patients. In addition, the population PK parameters of vancomycin in obese patients were compared with those in the general patient population analyzed under identical methodological conditions to characterize obesity-related alterations in vancomycin disposition.

## 2. Results

### 2.1. Patient Characteristics

**Figure 1:**
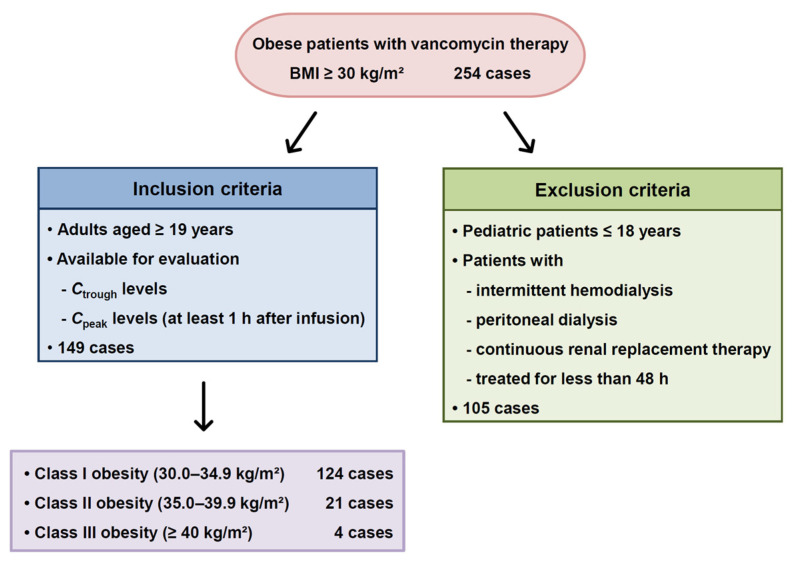
Flow diagram of patient selection in obese patients receiving vancomycin therapy. BMI, body mass index; *C*_peak_, peak plasma concentration; *C*_trough_, trough plasma concentration.

Among obese patients [body mass index (BMI) ≥ 30 kg/m^2^] treated at Ajou University Hospital during the 8-year study period (January 2010 to December 2017), a total of 254 cases (115 patients) who received vancomycin and underwent TDM were initially screened. Of these, 105 (44 patients) were excluded (Figure 1). The most common reason for exclusion was the use of intermittent hemodialysis, peritoneal dialysis, or continuous renal replacement therapy (CRRT), which accounted for 77 cases (73.3%; 34 patients). This was followed by 27 cases (25.7%) that did not meet the two-point blood sampling criteria, and one case (0.95%) that was excluded due to a blood sampling error.

The baseline characteristics are summarized in Table 1. The median [interquartile (IQR)] age was 53 (40–71) years, body weight was 88.7 (74.5–96.0) kg, and BMI was 31.3 (30.5–33.6) kg/m^2^, respectively. Most cases were categorized as obesity class I (30.0–34.9 kg/m^2^), comprising 124 cases (83.2%), followed by class II (35.0–39.9 kg/m^2^) with 21 cases (14.1%) and class III (≥40 kg/m^2^) with 4 cases (2.7%). The median serum creatinine concentration was 0.9 (0.7–1.1) mg/dL, and the median creatinine clearance was 118 (81.4–140) mL/min.

### 2.2. Distribution of AUC and C_trough_ Levels

The distributions of the AUC and *C*_trough_ values estimated using Bayesian software programs are presented in Table 2. The median (IQR) reference AUC (AUC_REF_) was 470 (372–578) mg·h/L. The median AUC obtained from a one-point blood sample (AUC_1p_) estimated using Capcil was 402 (293–524) mg·h/L, whereas that estimated using MwPharm was 440 (359–595) mg·h/L.

Although both AUC_1p_ estimates differed significantly from AUC_REF_, MwPharm produced estimates that were closer to the reference values and exhibited lower bias than Capcil (*p* < 0.01 for Capcil; *p* < 0.05 for MwPharm).

For MwPharm, no significant difference was observed between the AUC_1p_ and AUC obtained from two-point blood samples (AUC_2p_) estimates (*p* = 0.561).

In contrast, *C*_trough_ derived from a one-point blood sample (*C*_trough,1p_) for either Capcil or MwPharm was not significantly different from the reference *C*_trough_ (*C*_trough,REF_) value. Similarly, when MwPharm was used, no significant difference was observed between *C*_trough,1p_ and *C*_trough,2p_ estimates. These findings indicate that, while *C*_trough_ estimation is robust under reduced sampling conditions, AUC estimation based on single-point blood sampling exhibits greater variability.

### 2.3. Correlation Analysis

#### 2.3.1. Relationship Between C_trough,REF_ and AUC_REF_

**Figure 2:**
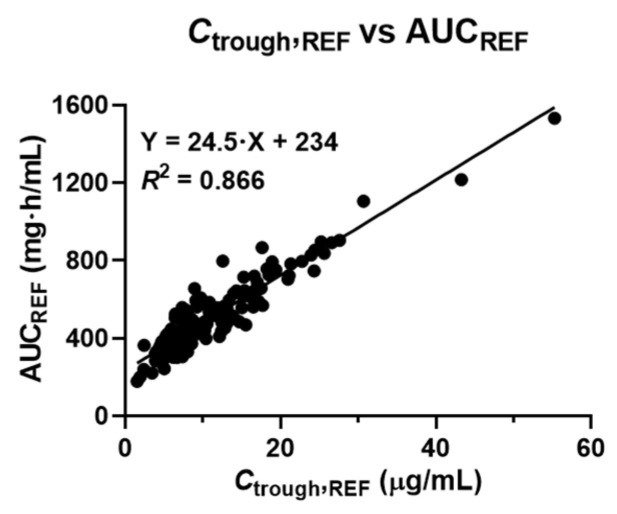
Scatter plot showing the relationship between *C*_trough,REF_ and AUC_REF_ in obese patients receiving vancomycin therapy. *C*_trough,REF_ and AUC_REF_ values were estimated for each patient using Capcil based on two-point blood sampling data. Linear regression analysis was performed to evaluate the correlation between *C*_trough,REF_ and AUC_REF_. AUC, area under the plasma concentration-time curve; *C*_trough_, trough plasma concentration; AUC_REF_, reference AUC; *C*_trough,REF_, reference *C*_trough_.

Figure 2 illustrates the relationship between *C*_trough,REF_ and AUC_REF_. A strong correlation was observed between *C*_trough,REF_ and AUC_REF_ (*R*^2^ = 0.866, *p* < 0.01). When *C*_trough,REF_ values were within the range of 15–20 μg/mL as recommended by the 2009 guideline, the corresponding AUC_REF_ values showed a median (IQR) of 657 (598–739) mg·h/L. Conversely, when AUC_REF_ values were within the target therapeutic range recommended by the 2020 guideline (400–600 mg·h/L), the corresponding *C*_trough,REF_ levels were lower, with a median of 10.0 (7.71–12.5) μg/mL, and were lower than those recommended in the previous guideline (Table 3).

#### 2.3.2. Relationship Between AUC_REF_ and AUC_1p_

**Figure 3:**
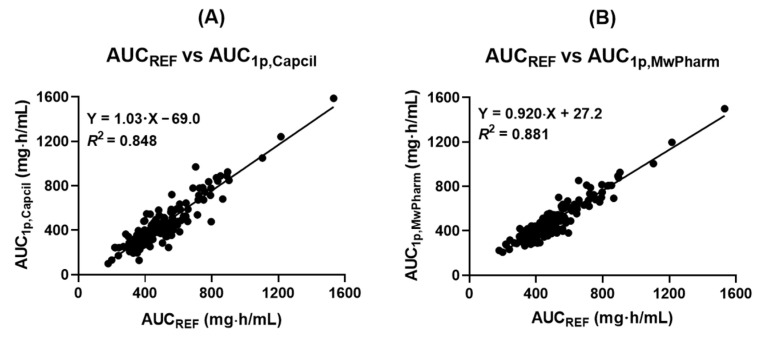
Scatter plot showing the relationship between (**A**) AUC_REF_ and AUC_1p,Capcil_ and between (**B**) AUC_REF_ and AUC_1p,MwPharm_ in obese patients receiving vancomycin therapy. AUC_REF_ values were estimated for each patient using Capcil based on two-point blood sampling data. AUC_1p,Capcil_ and AUC_1p,MwPharm_ values were estimated for each patient based on one-point blood sampling data using Capcil and MwPharm, respectively. Linear regression analysis was performed to evaluate the correlation between AUC_REF_ and AUC_1p,Capcil_ and between AUC_REF_ and AUC_1p,MwPharm_. AUC, area under the plasma concentration-time curve; AUC_REF_, reference AUC.

Correlation analyses between AUC_REF_ and AUC_1p_ estimates demonstrated significant associations for both Bayesian programs. MwPharm exhibited a higher coefficient of determination (Figure 3B, *R*^2^ = 0.881) than Capcil (Figure 3A, *R*^2^ = 0.848). Notably, Capcil-derived AUC_1p_ estimates demonstrated systematic underestimation relative to AUC_REF_, as indicated by a negative *y*-intercept (−69.0 mg·h/L) in regression analysis.

#### 2.3.3. Relationship Between AUC_1p_ and AUC_2p_ Estimated Using MwPharm

**Figure 4:**
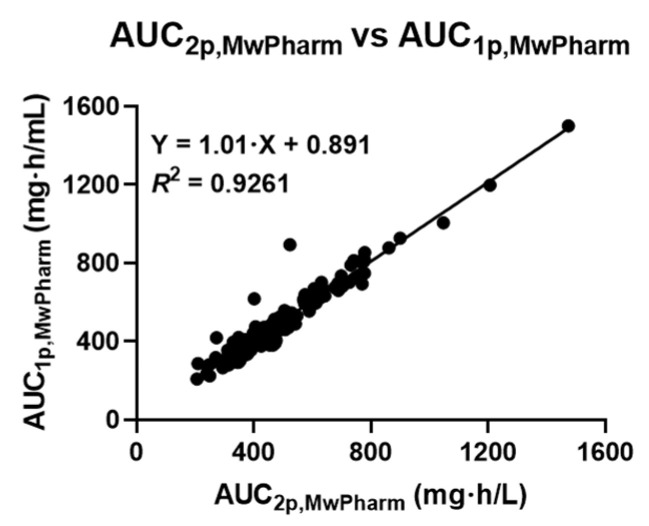
Scatter plot showing the relationship between AUC_2p,MwPharm_ and AUC_1p,MwPharm_ in obese patients receiving vancomycin therapy. AUC_1p,MwPharm_ and AUC_2p,MwPharm_ values were estimated for each patient based on one- and two-point blood sampling data, respectively, using MwPharm. Linear regression analysis was performed to evaluate the correlation between AUC_2p,MwPharm_ and AUC_1p,MwPharm_. AUC, area under the plasma concentration-time curve.

Figure 4 shows the correlation between the AUC_1p_ and AUC_2p_ estimates obtained using MwPharm. Notably, a strong and statistically significant correlation was observed (*R*^2^ = 0.926, *p* < 0.01).

### 2.4. Accuracy and Bias

**Figure 5:**
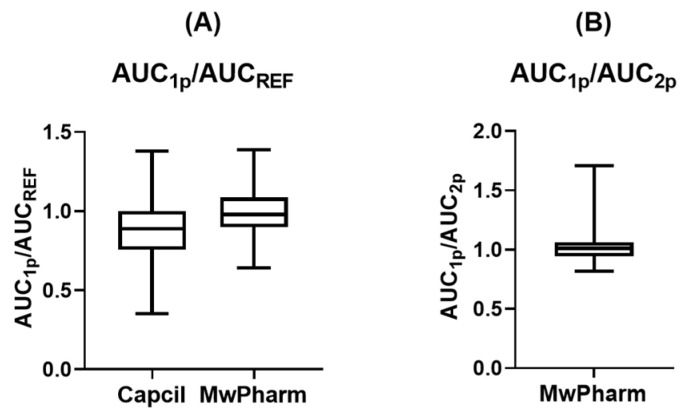
Distribution of AUC ratios according to estimation method. (**A**) AUC_1p_/AUC_REF_ ratios calculated using Capcil and MwPharm. (**B**) AUC_1p_/AUC_2p_ ratios calculated using MwPharm. Boxes represent the interquartile range (IQR), the horizontal line within each box indicates the median, and whiskers denote the minimum and maximum values. AUC_1p_ and AUC_2p_ values were estimated for each patient based on one- and two-point blood sampling data, respectively. AUC, area under the plasma concentration-time curve.

The accuracy of each Bayesian software program is summarized in Table 4. The median accuracy with IQR of AUC_1p_ estimated using Capcil relative to AUC_REF_ was 0.89 (0.75–1.00), whereas that estimated using MwPharm was 0.98 (0.90–1.08). These results indicate a greater deviation from the ideal accuracy value of 1.0 for Capcil compared with that of MwPharm. The distribution of accuracy values is shown in Figure 5. The AUC_1p_/AUC_REF_ ratios ranged from 0.35 to 1.38 for Capcil and from 0.64 to 1.39 for MwPharm, thereby indicating that Capcil exhibited a larger deviation than MwPharm. For *C*_trough,1p_ estimation, the median accuracies (IQR) of Capcil and MwPharm relative to *C*_trough,REF_ were 1.00 (0.99–1.01) and 0.98 (0.93–1.03), respectively, demonstrating relatively high accuracy for both programs. For MwPharm, the accuracies of AUC_1p_/AUC_2p_ and *C*_trough,1p_/*C*_trough,2p_ were generally similar, with median values of 1.01 and 1.0, respectively.

Bias analysis results are presented in Table 5. The AUC_1p_ estimates obtained using Capcil exhibited the greatest bias of 14.2% (5.29–26.4%) relative to AUC_REF_, whereas those obtained using MwPharm showed a lower bias of 9.88% (5.36–15.7%). For *C*_trough,1p_ estimates, consistent with the high accuracy observed, the degree of bias was relatively small at 1.14% (0.62–2.36%) for Capcil and 5.24% (2.63–9.27%) for MwPharm, compared with the bias observed for AUC_1p_ estimates. For MwPharm, the biases of AUC_1p_/AUC_2p_ and *C*_trough,1p_/*C*_trough,2p_ were generally low, with median values of 5.94% and 2.11%, respectively.

### 2.5. Population Pharmacokinetics

Population PK parameters in obese patients, estimated using two-point blood sampling and two Bayesian software programs, are presented in Table 6. Compared with the general patient population, obese patients exhibited lower weight-normalized *V*_d_ values (0.48–0.54 vs. 0.70 L/kg) and shorter elimination half-life (*t*_1/2_) (7.05–7.49 vs. 9.06 h) for both Bayesian software programs. In contrast, the total body clearance (CL) values were higher in obese patients (approximately 4.23–4.34 L/h) compared with 3.08 L/h in the general patient population. Correspondingly, elimination rate constant (*k*_e_) values were higher in obese patients than in the general patient population.

Table 7 shows the distribution of vancomycin dose and AUC_0–24h_ in obese patients and the general patient population. The proportion of patients with subtherapeutic exposure (AUC_0–24h_ < 400 mg·h/L) was higher in the obese group (32.2%) than in the general patient population (21.9%). This may be attributable to increased drug elimination observed in the obese population.

## 3. Discussion

This study evaluated the accuracy and bias of vancomycin AUC and *C*_trough_ estimates derived from one-point and two-point blood sampling using two Bayesian software programs in obese patients. Further, the pharmacokinetics of vancomycin in obese patients were compared with those in the general patient population. The principal findings can be summarized as follows: *C*_trough_ estimates derived from single-point blood sampling demonstrated high accuracy and low bias, regardless of the Bayesian software used. In contrast, AUC estimation based on single-point blood sampling showed considerable variability in obese patients and was strongly influenced by the choice of Bayesian software [22], with MwPharm providing estimates closer to the reference values and exhibiting lower bias than Capcil in this dataset. Additionally, vancomycin pharmacokinetics in obese patients differed substantially from those in the general patient population, characterized by a smaller *V*_d_, higher CL, and shorter *t*_1/2_.

The use of historical data reflects changes in clinical practice, as two-point sampling was more commonly performed prior to the widespread adoption of trough-only and AUC-guided monitoring strategies [11,13]. Accordingly, the use of historical data enabled a direct comparison of sampling-based AUC estimation methods, which are no longer routinely feasible in current clinical practice.

Vancomycin is a hydrophilic antibiotic with a relatively low to moderate *V*_d_, and its distribution is more closely related to total body water than to adipose tissue [2,11]. Although body weight influences vancomycin distribution, increases in body weight do not result in a proportional expansion of *V*_d_. Consistent with this PK principle, the present population PK analysis demonstrated lower weight-normalized *V*_d_ values in obese patients (approximately 0.48–0.54 L/kg) compared to the general patient population (approximately 0.70 L/kg). These findings align with previous studies reporting reduced weight-normalized *V*_d_ values in obese patients, typically ranging from 0.24 to 0.52 L/kg [18,19].

In addition to the altered distribution, obese patients in the present study exhibited higher CL values and shorter *t*_1/2_ values than the general patient population analyzed under identical methodological conditions. Median vancomycin CL values in obese patients were approximately 4.23–4.34 L/h (Table 6), thereby exceeding those observed in the general patient population (3.08 L/h). These findings suggest a more rapid vancomycin elimination in obese patients, which may necessitate shorter dosing intervals or higher total daily doses to maintain adequate exposure.

Importantly, despite demonstrating higher CL, obese patients in the present study received lower weight-based doses compared with the general patient population. Consequently, the proportion of patients with subtherapeutic exposure (AUC_0–24h_ < 400 mg·h/L) was higher in the obese group. These findings suggest that conventional mg/kg dosing strategies may not adequately account for the increased drug elimination observed in obesity, potentially leading to underexposure in this population. Therefore, individualized dosing strategies guided by PK assessment may be more appropriate than relying solely on total body weight. Notably, the observed CL values were lower than those reported in studies of extremely obese populations, where vancomycin CL ranged from approximately 5.4 to 9.0 L/h [19,23]. This discrepancy likely reflects differences in obesity severity and renal function across the study populations, and underscores the importance of population-specific PK evaluation rather than extrapolation from cohorts with extreme obesity.

The transition from *C*_trough_-based monitoring to AUC-guided TDM represents a major paradigm shift in vancomycin dosing. The 2009 consensus guidelines recommended *C*_trough_ levels of 15–20 μg/mL as a surrogate for achieving adequate AUC/MIC exposure [4]. However, subsequent studies have demonstrated that *C*_trough_ is an imperfect surrogate for AUC and may be associated with an increased risk of nephrotoxicity, without corresponding improvements in clinical efficacy [10,24]. Consequently, the 2020 revised guidelines currently recommend direct AUC-guided dosing, targeting an AUC_0–24h_/MIC of 400–600, assuming an MIC of 1 mg/L as determined by broth microdilution [11].

In line with these recommendations, AUC/MIC in the present study was calculated using a fixed MIC value of 1 mg/L. While this approach facilitates standardized PD evaluation, it does not account for variability in MIC values across individual patients and pathogens. Therefore, the use of a fixed MIC may influence the interpretation of AUC/MIC target attainment and its clinical implications, and this limitation should be considered when applying the present findings to clinical practice.

In the present study, *C*_trough,1p_ estimation demonstrated high accuracy and low bias relative to the reference values, regardless of the Bayesian software used. These results indicate that *C*_trough_ can be reliably estimated from a single-point blood sample in obese patients, thereby supporting its continued utility in practical TDM applications when AUC estimation is not feasible. In contrast, the AUC_1p_ estimation showed substantial variability, with a wide range of accuracy observed for both Capcil and MwPharm. This discrepancy highlights a fundamental limitation of AUC_1p_ estimation in obese patients, in whom altered pharmacokinetics may amplify the influence of model assumptions and prior distributions embedded in Bayesian software programs.

The findings of the present study differ from those of previous analyses conducted in general patient populations, in which AUC estimates derived from one- and two-point blood sampling were not significantly different across multiple Bayesian programs [25]. The emergence of significant discrepancies in the obese subgroup suggests that obese individuals represent a population that is particularly vulnerable to AUC misestimations when simplified sampling strategies are applied. These results are consistent with prior reports demonstrating improved target attainment with two-point blood sampling in obese patients and reinforce the importance of cautious interpretation of single-point AUC estimates in this population [23].

From a clinical perspective, the implementation of AUC-guided TDM poses logistic challenges, including additional blood sampling, specialized software, and trained personnel. Although two-point blood sampling remains the most reliable approach for accurate AUC estimation, it is not always feasible in routine clinical practice. In such situations, careful selection of the Bayesian software and awareness of its limitations are essential. Our findings suggest that when single-point blood sampling is unavoidable, MwPharm may provide AUC estimates more consistent with the reference values than Capcil under the conditions evaluated in this study. Nevertheless, clinicians should interpret the AUC_1p_ estimates with caution and consider confirmatory sampling when considering dose escalations.

Despite its strengths, this study also had several limitations. First, the retrospective design and single-center setting of this study may limit the generalizability of the findings, and multicenter studies would be valuable to validate these results across diverse patient populations and clinical settings, highlighting the need for external validation. In addition, the majority of patients were classified as having class I obesity, reflecting the epidemiological distribution of obesity in Korea, where class I obesity predominates, and the proportion of patients with more severe obesity (class II–III) remains relatively low [26]. Therefore, caution is warranted when extrapolating these findings to patients with more severe obesity [27,28].

Second, the PK models implemented in the evaluated software programs were not specifically developed for obese populations. As obesity is associated with altered PK characteristics, including changes in *V*_d_ and CL, the applicability of these models to obese patients may be limited. Accordingly, these findings should be interpreted as a comparison of model performance within two clinically available software platforms, rather than as a comprehensive assessment of all PK models applicable to obesity. Further studies incorporating obesity-specific models would help to better characterize model performance in this population.

Third, the AUC_REF_ in this study was derived using a data-driven fitting approach based on measured concentrations under routine clinical sampling conditions, rather than conventional two-point steady-state methods. Accordingly, the estimated AUC values should be interpreted within the context of this approach, particularly when comparing results across different PK models.

Fourth, the analysis was restricted to patients with available two-point blood samples, thereby potentially introducing a selection bias.

Fifth, individualized MIC values were not available for all patients, and AUC/MIC calculations were based on standard assumptions. Although AUC/MIC is clinically relevant, the primary objective of this study was to evaluate the accuracy of AUC estimation rather than AUC/MIC-based target attainment. Accordingly, the absence of patient-specific MIC values should be considered primarily in the interpretation of clinical implications rather than in the estimation of AUC itself.

Finally, potential confounding factors, such as comorbidities and concomitant use of nephrotoxic agents [10,29], were not systematically evaluated due to the retrospective nature of the study. These factors may have influenced vancomycin pharmacokinetics and should be considered when interpreting the findings [11]. Future studies incorporating a more comprehensive assessment of clinical covariates would further strengthen these findings.

Nonetheless, this study leveraged real-world clinical data and applied consistent analytical methods to both obese and general patient populations, thereby strengthening the validity of the comparative PK findings.

## 4. Materials and Methods

### 4.1. Study Subjects and Data Collection

This retrospective study included adult obese patients (body mass index [BMI] ≥ 30 kg/m^2^) who received intravenous vancomycin at Ajou University Hospital (Suwon, Republic of Korea) between January 2010 and December 2017. Clinical and demographic data, including age, sex, body weight, renal function parameters, vancomycin dosing history, blood sampling times, and vancomycin plasma concentrations, were obtained from electronic medical records.

Eligible patients were aged ≥19 years and underwent TDM during vancomycin therapy with the available pre-dose *C*_trough_ and post-dose peak plasma concentrations (*C*_peak_). Post-dose samples were collected at least 1 h after completion of vancomycin infusion to ensure post-distribution sampling [11,30]. Exclusion criteria included patients receiving intermittent hemodialysis, peritoneal dialysis, or CRRT, and those treated with vancomycin for less than 48 h.

Data were retrospectively collected from 2010 to 2017, a period during which two-point blood sampling (*C*_peak_ and *C*_trough_) was routinely performed at our institution, enabling a direct comparison of sampling-based AUC estimation methods.

The study protocol was approved (AJIRB-DB-2024-290, 19 June 2024) by the Institutional Review Board of the Ajou University Hospital (Suwon, Republic of Korea).

### 4.2. Vancomycin Dosing

Vancomycin dosing was performed according to institutional protocols based primarily on total body weight (TBW). In obese patients, TBW was used for both loading and maintenance dose calculations rather than ideal body weight (IBW). Loading doses, when administered, were typically 20–25 mg/kg based on TBW and were given at the discretion of the treating physician. Maintenance dosing was subsequently adjusted according to renal function and therapeutic drug monitoring results. During the study period, dose adjustments were primarily guided by trough concentrations, reflecting standard clinical practice at that time, whereas Bayesian estimation was applied retrospectively for the purposes of this analysis.

### 4.3. Study Design

This study aimed to evaluate the performance of Bayesian software programs in estimating vancomycin exposure in obese patients. The primary objective was to assess the accuracy and bias of vancomycin AUC and *C*_trough_ estimates derived from single-point blood sampling using estimates obtained from two-point blood sampling as reference values (AUC_REF_ and *C*_trough,REF_).

The second objective was to characterize the population PK parameters of vancomycin in obese patients using two-point blood sampling data and to compare these parameters with those obtained from a general patient population analyzed under identical methodological conditions. This approach aimed to minimize methodological variability and isolate the effects of obesity on vancomycin pharmacokinetics.

### 4.4. Data Analysis

#### 4.4.1. Bayesian Software Programs

Bayesian estimation of vancomycin exposure was performed using Capcil^®^ (ver 6.31, Simkin Inc., Gainesville, FL, USA) and MwPharm^++®^ (ver 2.3.1.89; Mediware, Prague, Czech Republic). Both programs incorporate patient-specific demographic and clinical variables, vancomycin dosing history, sampling time, and vancomycin plasma concentrations.

A two-compartment PK model was applied to MwPharm. Population PK parameters reported by Rodvold et al. [24] were used as Bayesian priors for MwPharm analysis. These priors were derived from adult patient populations and have been widely used in the Bayesian forecasting of vancomycin exposure. Capcil utilizes an internally implemented Bayesian framework based on population PK models embedded in the software.

#### 4.4.2. AUC Estimation

##### AUC_REF_

The AUC_REF_ was derived using a data-driven fitting approach based on measured plasma concentrations. Individual PK parameters were estimated using Capcil by fitting observed concentrations, without the use of Bayesian priors. The estimated parameters were subsequently applied to first-order PK equations to calculate CL and derive AUC. To account for non–steady-state conditions, the method proposed by Pai et al. [14] was applied. Accordingly, AUC_REF_ should be interpreted as a model-informed reference value rather than a directly observed ground truth.

##### AUC_1p_ and AUC_2p_

The AUC values estimated by Bayesian analysis using only *C*_trough_ from single-point blood samples were defined as AUC_1p_. The AUC values estimated using both *C*_peak_ and *C*_trough_ were defined as AUC_2p_. For MwPharm, which automatically calculates the AUC, the software-generated AUC values were used directly.

##### C_trough_ Estimation

*C*_trough_ estimates were categorized according to the number of blood samples (*C*_trough,1p_ and *C*_trough,2p_) and *C*_trough,REF_ and the Bayesian software applied. Additionally, *C*_trough,REF_ values were defined as those estimated using two-point blood samples.

##### Accuracy and Bias

The accuracy was evaluated by calculating the ratio of the estimated AUC to the AUC_REF_ (Equation (1)).

(1) $$Accuracy=\frac{AUC}{{AUC}_{REF}}$$

Bias was assessed as the percentage difference between the estimated AUC and AUC_REF_ to the AUC_REF_ (Equation (2)).

(2) $$\mathrm{Bias}\;(\%)=\frac{\left|AUC-{AUC}_{REF}\right|}{{AUC}_{REF}}\times 100$$

The same analytical approach was applied to the evaluation of *C*_trough_ estimates.

### 4.5. Population PK Analysis

Population PK analysis was performed using two-point blood sampling data obtained during routine TDM. Individual PK parameters were estimated using Bayesian posterior estimation implemented in Capcil and MwPharm. For MwPharm analysis, a two-compartment PK model was applied, and the population PK parameters reported by Rodvold et al. [31] were used as Bayesian priors. The evaluated PK parameters included *V*_d_, CL, *t*_1/2_, and *k*_e_. These parameters were derived from the posterior Bayesian estimates.

To assess the influence of obesity on vancomycin pharmacokinetics, PK parameters estimated in obese patients were compared with those obtained from a general patient population derived from a previously published clinical PK study conducted at the same institution, where vancomycin PK parameters were estimated using two-point blood sampling and Bayesian interpretation with Capcil [21]. In addition, vancomycin dose and AUC_0–24h_ distribution between obese patients and the general patient population were compared.

### 4.6. Statistical Analysis

Continuous variables were assessed for normality. Comparisons between the estimated and reference values were performed using paired *t*-tests or Wilcoxon signed-rank tests, as appropriate, based on data distribution. Further, linear regression analysis was conducted to evaluate the correlation between the estimated and reference values, expressed as the coefficient of determination (*R*^2^). For the population PK analysis, comparisons between obese patients and the general patient population were performed using the Mann–Whitney U test. For the analysis of AUC_0–24h_ distribution, the chi-square test was used to compare obese patients with the general patient population. A *p*-value < 0.05 was considered statistically significant. All statistical analyses were performed using SPSS version 25 (IBM Corp., Armonk, NY, USA). All data were presented as median with IQR ranges.

## 5. Conclusions

The pharmacokinetics of vancomycin in obese patients differ significantly from those in the general patient population, with a smaller *V*_d_, higher CL, and shorter *t*_1/2_. Although single-point blood sampling provides reliable *C*_trough_ estimates, AUC estimation based on single-point sampling is highly variable in obese patients and is influenced by the choice of Bayesian software. Two-point blood sampling remains preferable for accurate AUC-guided TDM in this population, and the cautious interpretation of single-point AUC estimates is warranted to optimize vancomycin dosing and minimize the risk of toxicity when simplified sampling strategies are employed.

## Figures and Tables

**Table 1:** Baseline demographic and clinical characteristics of obese patients.

Characteristic	Data
Number of cases (number of patients)	149 (71)
Age (year)	53 (40–71)
Male	74 (49.7%)
Height (cm)	162 (153–173)
Body weight (kg)	88.7 (74.5–96)
Ideal body weight (kg)	56.4 (45.5–68.7)
Body surface area (m^2^)	1.9 (1.7–2)
BMI (kg/m^2^)	31.3 (30.5–33.6)
Obesity class 1 (30.0–34.9)	124 cases (83.2%)
Obesity class 2 (35.0–39.9)	21 cases (14.1%)
Obesity class 3 (40.0–44.9)	4 cases (2.7%)
Serum creatinine (mg/dL)	0.9 (0.7–1.1)
Creatinine clearance (mL/min)	118 (81.4–140)
30.0–59.9	17 cases (11.4%)
60.0–89.9	26 cases (17.4%)
90.0–119.9	43 cases (28.9%)
≥120	63 cases (42.3%)
Creatinine clearance (mL/min/1.73 m^2^)	99.1 (78.1–126)

Data are presented as median with interquartile (IQR) range, or number (%). Obesity classes were defined according to BMI criteria. Creatinine clearance was calculated using actual body weight (Cockcroft–Gault equation) and is shown both as absolute values (mL/min) and body surface area–adjusted values (mL/min/1.73 m^2^). BMI, body mass index.

**Table 2:** AUC and *C*_trough_ values estimated by Bayesian software in obese patients.

	Parameters	Values	Program
AUC (mg·h/L)	AUC_REF_	470 (372–578)	
	AUC_1p_	402 (293–524) **	Capcil
		440 (359–595) *	MwPharm
	AUC_2p_	460 (369–573)	MwPharm
*C*_trough_ (μg/mL)	*C*_trough,_ _REF_	8.62 (6.35–13.5)	
	*C*_trough,1p_	9.13 (6.45–13.5)	Capcil
		8.56 (6.40–13.5)	MwPharm
	*C*_trough,2p_	8.44 (6.41–13.4)	MwPharm

Data are presented as median with interquartile (IQR) ranges. For the calculation of AUC_REF_, individualized PK parameters were estimated from two measured plasma concentrations using a data-driven fitting approach implemented in Capcil without incorporating Bayesian priors. The estimated parameters were subsequently applied to first-order PK equations to derive AUC, based on the method proposed by Pai et al. [14]. Comparisons between estimated and reference values or between values estimated from one- and two-point blood data for MwPharm were performed using paired *t*-tests. *, *p* < 0.05; **, *p* < 0.01. AUC, area under the plasma concentration–time curve; AUC_REF_, reference AUC; AUC_1p_ and AUC_2p_, AUC obtained from one- and two-point blood samples, respectively; *C*_trough_, trough plasma concentration; *C*_trough,REF_, reference *C*_trough_, *C*_trough,1p_ and *C*_trough,2p_, *C*_trough_ derived from one- and two-point blood samples, respectively.

**Table 3:** Distribution of AUC and *C*_trough_ according to therapeutic ranges in patients with MRSA infection.

PK/PD Parameters	Target Therapeutic Ranges	Corresponding PK/PD Parameters	Data
*C*_trough,REF_ (μg/mL)	15–20 ^a^	AUC_REF_ (mg·h/L)	657 (598–739)
AUC_REF_ (mg·h/L)	400–600 ^b^	*C*_trough,REF_ (μg/mL)	10.0 (7.71–12.5)

Data are presented as median with interquartile (IQR) ranges. ^a^ and ^b^ are based on the 2009 [4] and 2020 [11] guidelines, respectively. AUC, area under the plasma concentration-time curve; *C*_trough_, trough plasma concentration; AUC_REF_ and *C*_trough,REF_, reference AUC and *C*_trough_, respectively; MRSA, methicillin-resistant *Staphylococcus aureus*; PK, pharmacokinetic; PD, pharmacodynamic.

**Table 4:** Accuracy of estimated AUC and *C*_trough_.

PK/PD Target	Ratio_1p/REF_		Ratio_1p/2p_
	Capcil	MwPharm	MwPharm
AUC	0.89 (0.75–1.00)	0.98 (0.9–1.08)	1.01 (0.94–1.06)
*C*_trough_	1.00 (0.99–1.01)	0.98 (0.93–1.03)	1.00 (0.98–1.02)

Data are presented as median with interquartile (IQR) ranges. AUC, area under the plasma concentration-time curve; *C*_trough_, trough plasma concentration; Ratio_1p/REF_, ratio of AUC or *C*_trough_ estimated from one-point blood sampling to corresponding reference value; Ratio_1p/2p_, ratio of AUC or *C*_trough_ estimated from one-point blood sampling to that estimated from two-point blood sampling; PK, pharmacokinetic; PD, pharmacodynamic.

**Table 5:** Relative bias (%) of estimated AUC and *C*_trough_.

PK/PD Target	% Bias_1p/REF_		% Bias_1p/2p_
	Capcil	MwPharm	MwPharm
AUC	14.2 (5.29–26.4)	9.88 (5.36–15.7)	5.94 (2.85–10.23)
*C*_trough_	1.14 (0.62–2.36)	5.24 (2.63–9.27)	2.11 (0.89–3.73)

Data are presented as median with interquartile (IQR) ranges. AUC, area under the plasma concentration-time curve; *C*_trough_, trough plasma concentration; % Bias_1p/REF_, (one-point estimate − reference value)/reference value × 100; % Bias_1p/2p_, (one-point estimate–two-point estimate)/two-point estimate × 100; PK, pharmacokinetic; PD, pharmacodynamic.

**Table 6:** PK parameters of vancomycin in obese patients estimated by two Bayesian software programs compared with a general patient population.

Parameters	Obese Patients (*n* = 149)		General Patient Population ^a^ (*n* = 210)
	Capcil	MwPharm	
*V*_d_ (L)	41.0 (30.2–50.8)	44.4 (40.5–52.6) *	42.2 (37.5–50.2)
*V*_d_ (L/kg)	0.48 (0.34–0.62) **	0.54 (0.47–0.61) ***	0.70 (0.66–0.76)
CL (L/h)	4.23 (3.04–5.56) **	4.34 (3.04–5.83) ***	3.08 (1.97–4.10)
*t*_1/2_ (h)	7.49 (5.57–11.4) **	7.05 (5.81–10.0) ***	9.06 (7.01–15.0)
*k*_e_ (h^−1^)	0.093 (0.061–0.124) **	0.098 (0.069–0.119) ***	0.076 (0.046–0.099)

^a^, Data for the general patient population were obtained from a previous study performed by Kim et al. [21] using two-point blood samples with the Capcil software program. Data are presented as median with interquartile (IQR) ranges. Comparisons between obese patients and the general patient population were performed using the Mann–Whitney U test. *, *p* < 0.05; **, *p* < 0.01; ***, *p* < 0.001. *V*_d_, volume of distribution; CL, total body clearance; *t*_1/2_, elimination half-life; *k*_e_, elimination rate constant.

**Table 7:** Comparison of dose and AUC_0–24h_ distribution between obese patients and the general patient population.

	Obese Patients (*n* = 149)	General Patient Population (*n* = 210)	*p* Value ^†^
Dose (mg/kg/day)	23.5 (20.2–26.7)	30.6 (18.7–38.4)	
AUC_0–24h_ (mg·h/L)			0.091 ^†^
<400	48 (32.2%)	46 (21.9%)	
400–599	68 (45.6%)	111 (52.9%)	
≥600	33 (22.1%)	53 (25.2%)	

Data are presented as median with interquartile (IQR) ranges or number of cases (%). Data for the general patient population were obtained from a previous study performed by Kim et al. [21]. Data for both populations were estimated using two-point blood samples with the Capcil software program. ^†^ The chi-square test was performed for comparison of AUC_0–24h_ distribution. AUC_0–24h_, area under the plasma concentration-time curve from 0 to 24 h.

## Data Availability

All data supporting the findings of this study are included within the article.

## Abbreviations

The following abbreviations are used in this manuscript:

AUC	area under the plasma concentration–time curve
AUC_0–24h_	area under the plasma concentration–time curve from 0 to 24 h
AUC_1p_	AUC obtained from one-point blood sample
AUC_2p_	AUC obtained from two-point blood samples
AUC_REF_	reference AUC
BMI	body mass index
*C*_peak_	peak plasma concentration
*C*_trough_	trough plasma concentration
*C*_trough,1p_	*C*_trough_ derived from one-point blood sample
*C*_trough,2p_	*C*_trough_ derived from two-point blood samples
*C*_trough,REF_	reference *C*_trough_
CL	total body clearance
CRRT	continuous renal replacement therapy
IBW	Ideal body weight
IQR	interquartile
*k*_e_	elimination rate constant
MIC	minimum inhibitory concentration
MRSA	methicillin-resistant *Staphylococcus aureus*
PD	pharmacodynamic
PK	pharmacokinetic
*t*_1/2_	elimination half-life
TBW	total body weight
TDM	therapeutic drug monitoring
*V*_d_	volume of distribution
